# Adenosine A_2A_ Receptor Up-Regulation Pre-Dates Deficits of Synaptic Plasticity and of Memory in Mice Exposed to Aβ_1–42_ to Model Early Alzheimer’s Disease

**DOI:** 10.3390/biom13081173

**Published:** 2023-07-28

**Authors:** Cátia R. Lopes, António C. Silva, Henrique B. Silva, Paula M. Canas, Paula Agostinho, Rodrigo A. Cunha, João Pedro Lopes

**Affiliations:** 1 CNC-Center for Neuroscience and Cell Biology, University of Coimbra, 3004-504 Coimbra, Portugal; catiasofiarl@gmail.com (C.R.L.); antonio.carvalhodasilva@gmail.com (A.C.S.); hbdasilva@gmail.com (H.B.S.); canas.paula@gmail.com (P.M.C.); pmgagostinho@gmail.com (P.A.); jpplopes@gmail.com (J.P.L.); 2 Faculty of Medicine, University of Coimbra, 3000-370 Coimbra, Portugal

**Keywords:** adenosine, A_2A_ receptor, Alzheimer’s disease, memory, LTP, synapse

## Abstract

The intracerebroventricular (icv) injection of amyloid peptides (Aβ) models Alzheimer’s disease (AD) in mice, as typified by the onset within 15 days of deficits of memory and of hippocampal long-term potentiation (LTP) that are prevented by the blockade of adenosine A_2A_ receptors (A_2A_R). Since A_2A_R overfunction is sufficient to trigger memory deficits, we tested if A_2A_R were upregulated in hippocampal synapses before the onset of memory deficits to support the hypothesis that A_2A_R overfunction could be a trigger of AD. Six to eight days after Aβ-icv injection, mice displayed no alterations of hippocampal dependent memory; however, they presented an increased excitability of hippocampal synapses, a slight increase in LTP magnitude in Schaffer fiber-CA1 pyramid synapses and an increased density of A_2A_R in hippocampal synapses. A_2A_R blockade with SCH58261 (50 nM) normalized excitability and LTP in hippocampal slices from mice sacrificed 7–8 days after Aβ-icv injection. Fifteen days after Aβ-icv injection, mice displayed evident deficits of hippocampal-dependent memory deterioration, with reduced hippocampal CA1 LTP but no hyperexcitability and a sustained increase in synaptic A_2A_R, which blockade restored LTP magnitude. This shows that the upregulation of synaptic A_2A_R precedes the onset of deterioration of memory and of hippocampal synaptic plasticity, supporting the hypothesis that the overfunction of synaptic A_2A_R could be a trigger of memory deterioration in AD.

## 1. Introduction

Alzheimer’s disease (AD) is the most common memory pathology. It is neurochemically characterized by an abnormal production of amyloid peptides (Aβ) and an hyperphosphorylation of microtubule-associated tau protein [1]. One of the earliest alterations in AD evolution is a dysfunction and loss of synapses [2,3,4]—in particular, of glutamatergic synapses [5,6]—with aberrant patterns of synaptic plasticity in prominently afflicted regions, namely, in the hippocampus (e.g., [7,8]). However, the mechanisms triggering this synaptotoxicity, already present in mild cognitive impairment [9,10,11], considered a prodrome of AD, are still unknown.

The adenosine modulation system has emerged in recent years as a candidate target to alleviate the burden of AD. Adenosine is released in an activity-dependent manner [12,13] and is responsible for assisting in the encoding of information salience in neuronal networks through a combined action of inhibitory A_1_ receptors (A_1_R) and facilitatory A_2A_ receptors (A_2A_R) (reviewed in [14]). Both A_1_R and A_2A_R are mainly located in excitatory synapses in the cerebral and limbic cortices [15,16,17]. In particular, A_2A_R increase glutamate release [18,19] and bolster NMDA receptor function in the hippocampus [20,21,22] to selectively control synaptic plasticity (e.g., [23,24]) as well as synaptotoxicity (e.g., [24,25,26]). Notably, the pharmacological, genetic or optogenetic overfunction of A_2A_R is actually sufficient to deteriorate behavioral performance in tests of spatial reference memory [22,27,28,29], and, conversely, the pharmacological or genetic blockade of A_2A_R afford a general neuroprotection against brain damage (reviewed in [14,30,31]) and prevent memory dysfunction in different animal models of AD [8,22,25,32,33,34]. This is in notable agreement with human epidemiological findings showing that the regular intake of caffeinated coffee (caffeine is a selective antagonist of central adenosine receptors [35]) attenuates memory deficits and the onset of AD (e.g., [36,37,38]) and that A_2A_R polymorphisms are associated with the incidence of AD [39,40]. Importantly, in different models of neurodegeneration, A_2A_R undergo a gain of function, typified by a parallel upregulation of cortical A_2A_R (e.g., [8,22,24,25,26,29,33]) and an increased formation of ATP-derived adenosine within cortical synapses [41,42,43,44,45], which is selectively directed at the activation of A_2A_R [20,41,42,43,44,45]. Furthermore, in animal models of temporal lobe epilepsy [42] and of Parkinson’s disease [45], A_2A_R overfunction predates alterations of synaptic plasticity and of behavior that are characteristic of these diseases, prompting the hypothesis that A_2A_R upregulation might actually be a trigger of brain dysfunction resulting from aberrant synaptic plasticity [46].

We took advantage of a model of early AD based on the intracerebroventricular (icv) administration of a suspension of soluble β-amyloid peptides monomers and oligomers (Aβ_1–42_) to test if hippocampal A_2A_R upregulation was a precocious event, i.e., pre-dating hippocampal-dependent memory deficits. Previous studies have shown that the selective A_2A_R antagonist SCH58261 prevents Aβ-induced dysfunction of synaptic plasticity and of memory [25,41], and that there is time gap of at least 14 days between the icv administration of Aβ_1–42_ and the establishment of memory dysfunction [25]. This was exploited to test if A_2A_R density was increased in hippocampal synapses before the onset of memory dysfunction and if this was coupled to adaptive compensatory alterations of synaptic transmission and plasticity in the AD presymptomatic period. 

## 2. Materials and Methods

### 2.1. Animals

**Figure 1:**
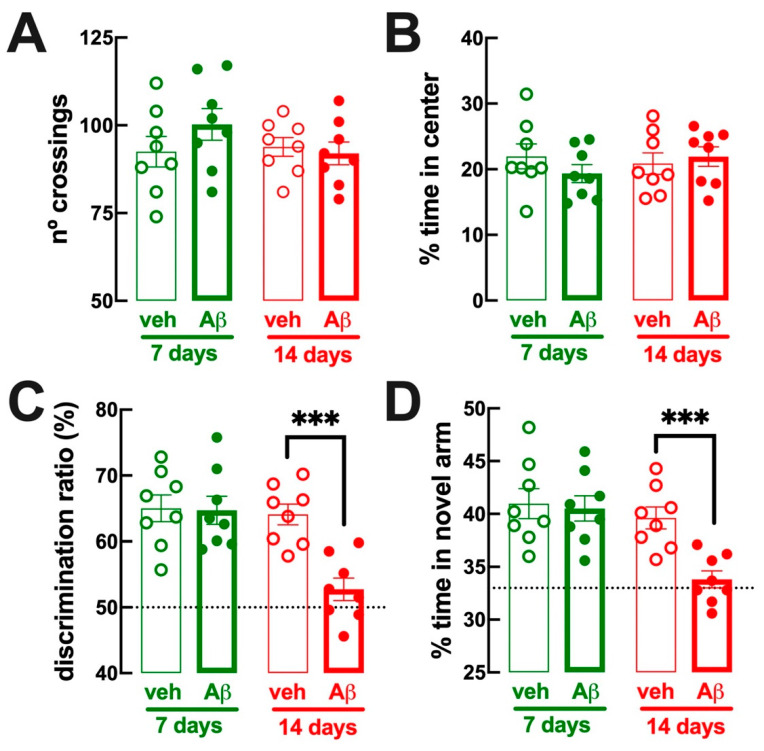
Mice challenged with an intracerebroventricular (icv) injection of β-amyloid (1–42) (Aβ) displayed a selective alteration of hippocampal-dependent spatial memory after 14–15 days, without behavioral alterations after 6–7 days. Compared to vehicle-icv-injected mice (veh, thin bars and open symbols), Aβ-icv-injected mice (Aβ, thick bars and filled symbols) analyzed at 6–7 days (green bars and symbols) or 14–15 days (red bars and symbols) after icv injections displayed a preserved spontaneous locomotion (**A**) and a preserved pattern of anxiety (**B**) in an open field arena. Memory performance in the object displacement test (**C**) and in the modified Y-maze test (**D**) was preserved after 6–7 days and deteriorated at 14–15 days. Data are mean ± SEM; *n* = 8 mice per group; *** *p* < 0.001, Student’s *t* test with Welsh correction.

**Figure 2:**
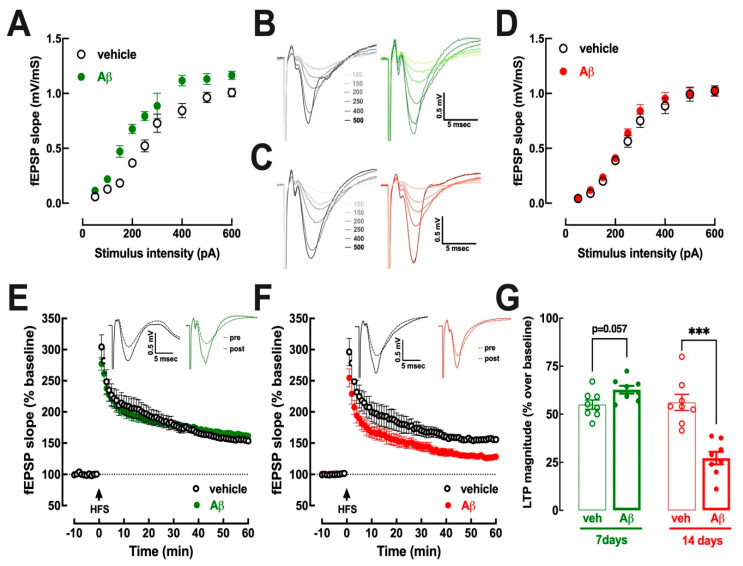
Hippocampal slices collected from mice during the presymptomatic period 7–8 days after an intracerebroventricular (icv) injection of β-amyloid 1–42 (Aβ-icv) displayed an hyperexcitability that was converted to a decreased pattern of synaptic plasticity upon onset of memory deficits at 15–16 days after Aβ-icv. At 7–8 days after Aβ-icv, and when compared to vehicle-icv injected mice (black open symbols/lines), Aβ-icv mice (filled green symbols/lines) displayed an hyperexcitability assessed by the shift to left of the input/output curve of the field excitatory post-synaptic potentials (fEPSP) slope (**A**,**B**) and a tendency towards an increased magnitude of long-term potentiation (LTP) triggered by a high-frequency stimulation train (HFS, 100 Hz for 1 s) in Aβ-icv mice (**E**,**G**). In contrast, in the symptomatic phase, there was no difference in the input/output curve between control and Aβ-icv mice (filled red symbols/lines) (**C**,**D**); however, LTP magnitude was lower in Aβ-icv mice (**F**,**G**). Data are mean ± SEM of 8 mice per group, the same as in Figure 1; *** *p* < 0.001 between indicated groups using Student’s *t* test with Welsh correction.

**Figure 3:**
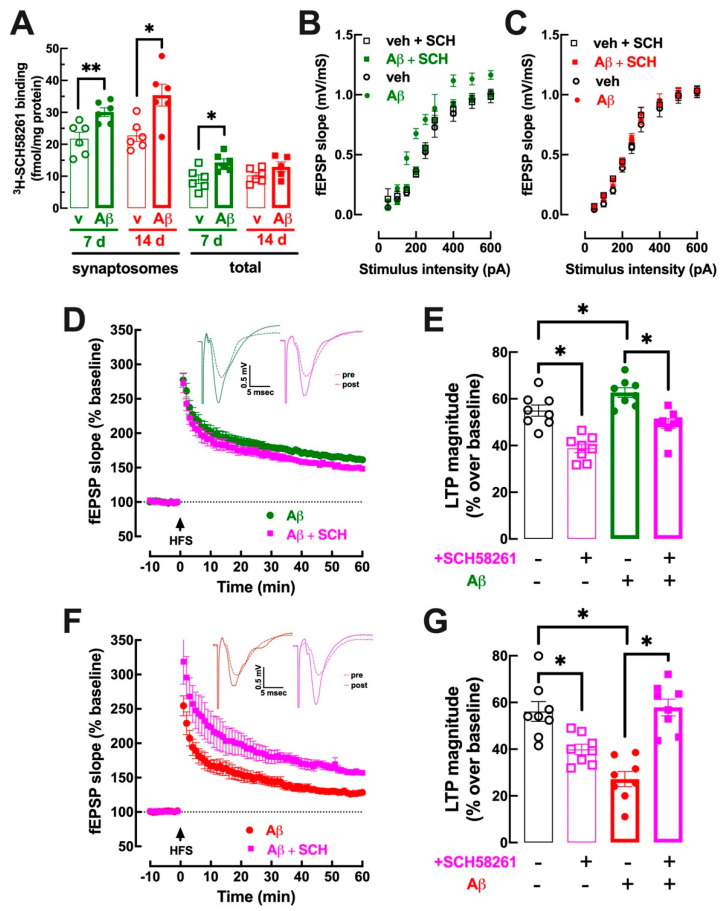
A_2A_R are upregulated from the presymptomatic phase of AD onward and control both hippocampal hyperexcitability in the presymptomatic phase and LTP deficits upon onset of memory deficits. (**A**) Binding of the A_2A_R antagonist ^3^H-SCH58261 (2 nM) in vehicle-icv mice (v, open symbols and thin bars) and in Aβ-icv mice (Aβ, filled symbols and thick bars) in hippocampal synaptosomal or total membranes at 7–8 days (green) or 15–16 days (red) after icv injections. Data are mean ± SEM of 6 mice per group; * *p* < 0.05 and ** *p* < 0.01 between indicated groups using Student’s *t* test with Welsh correction. (**B**) The addition of SCH58261 (50 nM) to hippocampal slices prevented the hyperexcitability in Aβ-icv mice and had no effect in vehicle-icv mice (veh) at 7–8 days, whereas SCH58261 was devoid of effects on hippocampal excitability in Aβ-icv and vehicle-icv mice at 15–16 days (**C**). SCH58261 decreased LTP magnitude triggered by a high-frequency stimulation train (HFS, 100 Hz for 1 s) in vehicle-icv-treated mice and normalized LTP magnitude in Aβ-icv both in the presymptomatic (**D**,**E**) and symptomatic phase (**F**,**G**). The vehicle-icv and Aβ-icv groups in Figure 3 are the same as in Figure 2. Data in (**B**–**G**) are mean ± SEM of 8 mice per group; * *p* < 0.05 between indicated groups using Tukey’s test after a two-way ANOVA.

We used adult C57bl/6j mice, 3 months of age (24.2 ± 1.7 g) and of both sexes (20 females and 12 males for the data presented in Figure 1 and Figure 2; 14 females and 18 males in the groups treated with the A_2A_R antagonist in Figure 3, with similar number of mice from each sex in each of the two groups), obtained from Charles River (Barcelona, Spain). We did not carry out a de novo power analysis, since we relied on our previous experience of the variability associated with the model used and drugs tested to pre-define the sample size as 8 mice per group in the behavioral and electrophysiology experiments and a minimum of 6 mice per group in the binding experiments in order to be able to detect 20% changes with 95% confidence. Mice were housed under controlled temperature (23 ± 1 °C) and a 12 h light/dark cycle, with free access to food and water. Mice were handled following European Community guidelines (EU Directive 2010/63/EU) and the Portuguese law on animal care (1005/92), and all procedures were approved by the Ethical Committee of the Center for Neuroscience and Cell Biology of Coimbra (ORBEA-128/2015). 

### 2.2. Intracerebroventricular Injection of Aβ to Model AD

The Aβ_1–42_ peptide fragment was purchased from Bachem (Bubendorf, Germany) and dissolved in water to obtain a solution mostly composed of small-molecular-weight Aβ oligomers [25,47] with a final concentration of 2.25 mg/mL. Mice were subjected to stereotaxic surgery for unilateral intracerebroventricular (icv) injection alternatively in the right or left hemisphere (dorsoventral: −2.00 mm; anteroposterior: −0.58 mm; lateral: ±1.13 mm) of Aβ (single dose of 2 nmol of Aβ_1–42_ in 4 µL) or vehicle (water, which caused no behavioral or neurochemical effects, similarly to the administration of scrambled Aβ_42–1_ (see [25])) under anesthesia with avertin, as previously performed [6,25,45]. This dose of Aβ_1–42_ translates into levels of 5–30 pmol Aβ_1–42_ within the hippocampus, causing synaptic alterations and selective reference memory dysfunction after 14 days without evidence of cellular damage [25], thus constituting a model of early AD, which has been previously used by different groups (e.g., [25,48,49]).

Behavioral analysis was performed before the onset of memory deficits (6–7 days after Aβ-icv injection) or when memory deficits became evident (14–15 days after Aβ-icv). Mice in the two groups were sacrificed in pairs (1 vehicle- and 1 Aβ-treated) between days 7–8 or days 15–16 after Aβ-icv injections by decapitation after deep anesthesia with 2-bromo-2-chloro-1,1,1-trifluoroethane (halothane from Sigma-Aldrich, Lisbon, Portugal; no reaction to handling or tail pinch, while still breathing).

### 2.3. Behavioral Analysis

All behavior tests were carried out as previously described [8,24,33,41] from 9 AM until 5 PM by experimenters who were unaware of drug treatments in a sound-attenuated room maintained at 21–23 °C and 50–60% humidity with red lightening (8 lux light intensity) and visual cues on the walls, to which the animals were previously habituated for at least 1 h before beginning behavioral tests. The tests were video-recorded and analyzed with the ANY-maze Video Tracking Software (version 6.18; Stoelting Europe, Dublin, Ireland). The apparatuses were cleaned with 10% ethyl alcohol to remove odors after testing each mouse.

Locomotion and exploratory behavior were monitored in the morning of either day 6 or day 14 after Aβ-icv injections, using an open-field arena, where each mouse was placed in the center of the open field to record during 10 min the following variables: number of peripheral squares (adjacent to the walls) crossed (peripheral locomotion); number of central squares (away from the walls) crossed (central locomotion); and total locomotion (peripheral locomotion plus central locomotion).

Hippocampal-dependent memory was evaluated using the object displacement test, carried out in the afternoon of either day 6 or day 14 after icv injections. Mice were exposed to two identical objects in the same open field apparatus in which they were habituated and were allowed to explore for 5 min the objects fixed in opposite corners 5 cm away from walls and 25 cm apart from each other. In a test trial carried out 2-h later, mice were again placed for 5 min in the open field arena, except that one of the objects was moved to a novel position. Memory performance was quantified with an object displacement index defined as the ratio between the time exploring the object in the novel location over the total time exploring both objects. Exploration of an object is defined as directing the nose to the object at a distance equal to or less than 1 cm from the object and/or touching it with the nose; rearing on to object was not considered exploratory behavior.

Spatial memory was further evaluated using a 2-trials Y-maze paradigm on the morning of either day 7 or day 15 after icv injections. The test consisted of two sessions of 8 min duration separated by a 90-min inter-trial interval. During the first session, the mouse was placed at the end of one arm and allowed to explore the two available arms since the third arm (the novel arm) was blocked by a guillotine door. During the second session, the ‘novel’ arm was open, and the mouse was placed in the start arm and allowed to explore the three arms. Memory performance was evaluated by measuring the time spent exploring the ‘novel’ arm compared to the exploration of the other two arms. An entry into an arm was defined as placement of all four paws into the arm.

### 2.4. Electrophysiological Recordings

Following sacrifice, the mouse brain was quickly removed and placed in ice-cold, oxygenated (95% O_2_, 5% CO_2_), artificial cerebrospinal fluid (ACSF; in mM: 124.0 NaCl, 4.4 KCl, 1.0 Na_2_HPO_4_, 25.0 NaHCO_3_, 2.0 CaCl_2_, 1.0 MgCl_2_, 10.0 glucose). One hemisphere was used for electrophysiological experiments and the other hemisphere was used for receptor binding studies (in 6 out of the 8 mice per group). Slices (400 μm-thick) from the dorsal hippocampus were cut transverse to the long axis of the hippocampus with a McIlwain tissue chopper (Mickle Laboratory Engineering Co., Surrey, UK) and maintained for at least 1 h prior to recording in a holding chamber with oxygenated ACSF at room temperature. Slices were then transferred to a submerged recording chamber and superfused at 3 mL/min with oxygenated ACSF kept at 30.5 °C. Electrophysiological recordings of field excitatory postsynaptic potentials (fEPSP) were carried out as previously described (e.g., [24,33,35,41]) with the recording electrode, filled with 4 M NaCl (2–5 MΩ resistance), placed in the CA1 stratum radiatum targeting the distal dendrites of pyramidal neurons and the stimulating bipolar concentric electrode placed in the proximal CA1 stratum radiatum. Rectangular pulses of 0.1 ms were delivered every 20 s through a Grass S44 or a Grass S48 square pulse stimulator (Grass Technologies, Singapore). After amplification (ISO-80, World Precision Instruments, Singapore), the recordings were digitized (Pico ADC-42, Pico Technologies Ltd., St. Neots, UK), averaged in groups of 3 and analyzed using the WinLTP version 2.10 software [50]. 

Input/output (I/O) curves, in which the percentage of the maximum fEPSP slope was plotted versus stimulus intensity, were first performed in all slices to evaluate changes in basal synaptic transmission and to determine the adequate level of electrical stimulation required to trigger between 50–60% of maximal fEPSP response, which was used for the remainder of the experimental protocol. Long-term potentiation (LTP) was induced by a high-frequency stimulation train (100 Hz for 1 s). LTP was quantified as the percentage change between the average slope of the 10 fEPSPs between 50 and 60 min after LTP induction in relation to the average slope of the fEPSPs measured during the 10 min that preceded LTP induction. The effect of SCH58261 (50 nM) on LTP was assessed by comparing LTP magnitude in the absence and presence of SCH58261 in experiments carried out in different slices from the same animal.

### 2.5. Membrane Binding Assays

Following sacrifice, one hippocampus of each mouse was dissected and homogenized in ice-cold sucrose solution (0.32 M D-sucrose; 1 mM EDTA-Na; 10 mM HEPES; 0.015 mM bovine serum albumin; pH 7.4 at 4 °C) for subsequent preparation of synaptosomes and total membranes, as previously described (e.g., [16,17]). The synaptosomal fraction and the total membrane fraction were each resuspended in 300 µL of a pre-incubation solution (50 mM Tris, 1 mM EDTA, 2 mM EGTA, pH 7.4) to determine their protein content with the Bio-Rad protein assay (Bio-Rad, Amadora, Portugal) and stored at −80 °C until used for membrane binding assays. 

Binding assays to estimate A_2A_R density in synaptosomal membranes were carried out as previously described (e.g., [8,24,26,33]). Briefly, the synaptosomes were lysed in a Tris/Mg solution (50 mM Tris, 10 mM MgCl_2_, pH 7.4) and pelleted. Both synaptosomal membranes and total membranes were incubated for 30 min at 37 °C with adenosine deaminase (2 U/mL; Roche Molecular Biochemicals, Indianapolis, IN, USA) to remove endogenous adenosine. After centrifugation at 24,000× *g* for 15 min at 4 °C, the pellets were resuspended in 600 µL of Tris/Mg solution with 4 U/mL of adenosine deaminase. A_2A_R binding density was determined with 217–345 μg of synaptosomal membrane protein exposed during 1 h at room temperature to a single supra-maximal concentration (2 nM) of the selective antagonist ^3^H-SCH58261 (specific activity of 77 Ci/mmol, prepared by GE Healthcare Life Sciences and generously offered by E. Ongini, Schering-Plough, Italy) in a final volume of 200 µL. The binding reactions were stopped by addition of 5 mL of ice-cold Tris/Mg and vacuum filtration through glass fiber filters (Whatman GF/C filters, GE Healthcare Life Sciences, Carnaxide, Portugal), followed by a washing step with 5 mL of ice-cold Tris/Mg. Filters were then placed in vials with 2 mL of scintillation liquid (AquaSafe 500Plus; Zinsser Analytic GMBH, Eschborn, Germany) to measure radioactivity in a 2900TR Tricarb β-counter (PerkinElmer, Lisbon, Portugal) after at least 12 h. Specific binding was expressed as fmol/mg of protein and determined via subtraction of the non-specific binding, measured in the presence of the mixed A_1_/A_2A_ receptor antagonist XAC (Sigma-Aldrich, Lisbon, Portugal) at a concentration (12 µM) over 1000 times higher than that of the radioligand. Total binding measurements were conducted in triplicate, and nonspecific binding in duplicate. Negative controls in hippocampal membranes of A_2A_R knockout mice have previously ensured the selectivity of the tested concentration of ^3^H-SCH58261 [51].

### 2.6. Statistical Analyses

The values are presented as mean ± S.E.M. with the number of determinations (*n*, preparations from different mice). A Grubbs’ test was first used to detect putative outliers. The comparison of two experimental conditions was performed using a two-tail Student’s *t* test with Welsh correction. Otherwise, statistical analysis was performed by two-way analysis of variance (ANOVA) followed by a Tukey’s multiple comparison post hoc test. *p* < 0.05 was considered to represent statistical significance. Statistical analysis was performed using GraphPad Prism software (version 6.0; GraphPad Software, La Jolla, CA, USA).

## 3. Results

### 3.1. Memory Deficits Are Present at 14 Days but Not at 7 Days after Aβ Administration

The Aβ-icv mouse model of AD is characterized by selective hippocampal-dependent memory deficits emerging 12 days after Aβ-icv administration without motor or emotional alterations [25,41]. Accordingly, compared to vehicle-treated mice, we now observed that 6–7 days after Aβ-icv administration, mice were devoid of alteration of (i) spontaneous locomotion in the open field test (number of line crossing: 92.5 ± 4.3 for vehicle and 100.3 ± 4.5 for Aβ-icv, *n* = 8, *p* = 0.236 with Student’s *t* test (Figure 1A)); (ii) emotional-like behavior assessed as the percentage of time spent in the more aversive central zone of the open field arena (22.0 ± 1.9% for vehicle and 19.3 ± 1.4% for Aβ-icv, *n* = 8, *p* = 0.280 with Student’s *t* test (Figure 1B)); (iii) hippocampal-dependent memory performance in the object displacement test (discrimination ratio: 65.0 ± 2.0 for vehicle and 64.7 ± 2.1 for Aβ-icv, *n* = 8, *p* = 0.915 with Student’s *t* test (Figure 1C)); and (iv) memory performance in the modified Y-maze test (percentage time in the new arm: 41.0 ± 1.4% for vehicle and 40.5 ± 1.2% for Aβ-icv, *n* = 8, *p* = 0.806 with Student’s *t* test (Figure 1D)). In contrast, 14–15 days after Aβ-icv administration, mice displayed a selective deficit of hippocampal-dependent memory performance both in the object displacement test (discrimination ratio: 64.1 ± 1.6 for vehicle and 52.7 ± 1.7 for Aβ-icv, *n* = 8, *p* < 0.001 with Student’s *t* test (Figure 1C); with similar discrimination ratio during the training period: 50.7 ± 1.0 for vehicle and 50.3 ± 1.0 for Aβ-icv, *n* = 8, *p* = 0.778 with Student’s *t* test), as well as in the modified Y-maze test (percentage time in the new arm: 39.6 ± 1.0% for vehicle and 33.8 ± 0.8% for Aβ-icv, *n* = 8, *p* < 0.001 with Student’s *t* test (Figure 1D)), without evident alterations of locomotion (number of line crossings: 93.9 ± 2.7 for vehicle and 92.0 ± 3.3 for Aβ-icv, *n* = 8, *p* = 0.663 with Student’s *t* test (Figure 1A)) or anxiety in the open field test (percentage time in the center: 20.9 ± 1.6% for vehicle and 21.9 ± 1.5% for Aβ-icv, *n* = 8, *p* = 0.638 with Student’s *t* test (Figure 1B)). Thus, Aβ-icv administration only triggers a selective hippocampal-dependent memory deficit at 14–15 days, without evident behavioral modification 6–7 days after Aβ-icv administration.

### 3.2. Hippocampal Excitability Increases at 7–8 Days and Decreases at 15–16 Days after Aβ Administration

In spite of the absence of behavioral alteration at days 6–7 after Aβ-icv, there were alterations of synaptic excitability in hippocampal slices of these mice. Thus, the recording of synaptic transmission in Schaffer fibers-CA1 pyramid synapses, assessed as field excitatory synaptic potentials (fEPSP), revealed an increase in the input/output (I/O) curves in slices of mice at days 7–8 after Aβ-icv compared to vehicle-treated controls (Figure 2A,B). This increased excitability is likely to be a transient compensatory feature since it disappeared in slices of mice at days 15–16 after Aβ-icv, which displayed I/O superimposable to these of vehicle-treated mice (Figure 2C,D).

As shown in Figure 2E,G, synaptic plasticity, assessed as the magnitude of long-term potentiation (LTP), displayed a tendency for an increase (*p* = 0.057, Student’s *t* test) in slices collected at days 7–8 after Aβ-icv (62.7 ± 2.4% over baseline, *n* = 8) compared to vehicle-treated controls (55.0 ± 2.4% over baseline, *n* = 8). This tendency for an increased LTP magnitude observed before the onset of memory deficits reverted to a decreased LTP magnitude at the onset of memory deficits (Figure 2F,G), as previously reported [41]. In fact, in slices collected at days 15–16 after Aβ-icv, LTP magnitude (27.2 ± 3.2% over baseline, *n* = 8) was lower (*p* < 0.001, Student’s *t* test) than LTP magnitude in slices from vehicle-treated mice (56.1 ± 4.2% over baseline, *n* = 8). 

### 3.3. Early Upregulation of Synaptic A_2A_R Is Responsible for Abnormal Hippocampal Function at 7–8 Days and at 15–16 Days after Aβ Administration

Since A_2A_R activation mimics [22,27,28] and A_2A_R blockade with SCH58261 prevents Aβ-icv-induced synaptic and memory deficits [25,41,52], and A_2A_R are up-regulated before the onset of some brain-induced dysfunction [26,45], we then tested if the density of A_2A_R in hippocampal synapses increased before the onset of synaptic and memory deficits triggered by Aβ-icv. As shown in Figure 3A, the binding density of a supramaximal but selective concentration (2 nM) of the A_2A_R antagonist ^3^H-SCH58261 [51] was larger (*p* < 0.008, Student’s *t* test) in hippocampal synaptosomal (i.e., synaptic) membranes from Aβ-icv-treated mice (30.1 ± 1.4 fmol/mg protein, *n* = 6) than from vehicle-treated mice at 7–8 days (21.8 ± 2.0 fmol/mg protein, *n* = 6), i.e., when memory performance was not yet modified (Figure 1C,D). In accordance with the previously described enrichment of A_2A_R in hippocampal synapses [17], the binding density of A_2A_R in synaptosomal membranes was higher (*p* = 0.006, Student’s *t* test) than in total membranes (9.1 ± 1.4 fmol/mg protein, *n* = 6) (Figure 3A, comparing first to fifth column from the left). However, still, in total hippocampal membranes, A_2A_R density was higher (*p* = 0.015, Student’s *t* test) 7–8 days after Aβ-icv administration 14.3 ± 1.1 fmol/mg protein, *n* = 6) compared to vehicle-treated mice (9.1 ± 1.4 fmol/mg protein, *n* = 6) (Figure 3A). When Aβ-induced memory deficits became evident, 15 days after Aβ-icv, A_2A_R density was still larger (*p* = 0.012, Student’s *t* test) in synaptosomal membranes from Aβ-icv treated (35.4 ± 3.4 fmol/mg protein, *n* = 6) compared to vehicle-treated mice (22.8 ± 1.8 fmol/mg protein, *n* = 6) (Figure 3A), but there was no significant difference (*p* = 0.175, Student’s *t* test) between the binding density of A_2A_R in total membranes from Aβ-icv-treated (13.0 ± 1.5 fmol/mg protein, *n* = 5) compared to vehicle-treated mice (10.2 ± 0.9 fmol/mg protein, *n* = 6) (Figure 3A).

We next investigated if A_2A_R blockade would be able to prevent the hyperexcitability observed in the hippocampus in the presymptomatic period, i.e., 7–8 days after Aβ-icv injection. The administration of a supramaximal concentration of SCH58261 (50 nM) [20] prevented the increased synaptic efficiency in the hippocampus 7–8 days after Aβ-icv administration, bringing the I/O curve to a profile nearly superimposable to that observed in vehicle-treated mice ((Figure 3B); note that the vehicle-icv and Aβ-icv groups in Figure 3B are the same as in Figure 2A). Moreover, the tendency for an increased LTP magnitude observed in hippocampal slices collected 7–8 days after Aβ-icv administration was eliminated by 50 nM SCH58261 ((Figure 3D); note that the vehicle-icv and Aβ-icv groups in Figure 3D,E are the same as in Figure 2C–E); thus, in slices collected 7–8 days after Aβ-icv administration, LTP magnitude was 62.7 ± 2.4% over baseline (*n* = 8) in the absence and was significantly lower (*p* < 0.001, Student’s *t* test) in the presence of SCH58261 (49.4 ± 2.1% over baseline, *n* = 8). A Tukey’s multiple comparison test after a two-way ANOVA (effect of Aβ: F_1,28_ = 18.82, *p* < 0.001; effect of SCH58261: F_1,28_ = 48.61, *p* < 0.001) revealed no significant difference between LTP magnitude in vehicle-treated and Aβ-icv-treated mice in the presence of SCH58261 (*p* = 0.291), although SCH58261 also decreased LTP magnitude in saline-treated mice (*p* < 0.001, Student’s *t* test), as previously reported (e.g., [26,35,41]).

The normalizing effect of SCH58261 in slices collected 15–16 days after Aβ-icv was similar to that previously described [41,53]. Thus, SCH58261 (50 nM) reverted (*p* < 0.001, Student’s *t* test) the Aβ-induced decrease in LTP magnitude (27.2.4 ± 3.2% over baseline, *n* = 8, in the absence and 57.9 ± 3.6% over baseline, *n* = 8, in the presence of SCH58261) ((Figure 3F,G); note that the vehicle-icv and Aβ-icv groups in Figure 3F,G are the same as in Figure 2D,E). A Tukey’s multiple comparison test after a two-way ANOVA (effect of Aβ: F_1,28_ = 6.61, *p* = 0.018; effect of SCH58261: F_1,28_ = 4.52, *p* = 0.043) revealed no significant differences of LTP magnitude between vehicle- and Aβ-icv-treated mice in the presence of SCH58261 (*p* = 0.983), although SCH58261 also decreased LTP magnitude in saline-treated mice (*p* = 0.011), as previously reported (e.g., [26,35,41]).

## 4. Discussion

The present study shows that an increased density and functional impact of A_2A_R in the hippocampus predates the Aβ-icv-induced deterioration of synaptic plasticity and of reference memory that are characteristic of this mouse model of AD. Thus, it was shown that the activity of these A_2A_R—with increased density in the presymptomatic period of AD that is maintained throughout the phenotypic expression of synaptic and memory deficits characteristic of early AD—is required both for the adaptive hyperexcitability before memory deficits as well as for the expression of synaptic and memory deficits present at the onset of symptomatic AD. 

We first characterized experimental conditions indicative of a presymptomatic period of AD, where an increase in hippocampal excitability was observed at 7–8 days after Aβ-icv, without any evident alteration of memory-related behavioral performance. This is in agreement with several studies reporting an initial hyperexcitability in the hippocampus in early AD and pre-dating the onset of memory deficits (reviewed in [54,55,56]), as well as with the presence of subclinical epileptiform activity in the AD prodrome [57,58] and its association with the onset of AD [59]. This initial hippocampal hyperexcitability is linked to a deregulation of the inhibitory GABAergic network [60,61,62], involving a parallel alteration of the excitability of different interneurons [60,61,63] as well as an altered astrocytic support [64,65]. Importantly, this initial hippocampal hyperexcitability may contribute to the subsequent deterioration of information processing in hippocampal networks and the onset/aggravation of memory symptoms of AD [54,55,56], as heralded by the ability of drugs refraining from excessive excitation, such as levetiracetam, to improve performance on spatial memory tasks in early AD [66]. 

The exploitation of this tentative mouse model of presymptomatic AD then allowed us to conclude that A_2A_R undergo an early upregulation and seem to be paramount to controlling the increased excitability during this prodrome-like period in this Aβ-icv model of AD. In fact, we observed an increased density of A_2A_R in the synapses, where cortical A_2A_R are most abundant [17], but also in total membranes that mostly include non-synaptic and astrocytic membranes. Moreover, we showed that a selective A_2A_R antagonist, SCH58261 [51], abrogated the hippocampal hyperexcitability present before the onset of memory deficits in this Aβ-icv model of AD. Although the precise mechanism underlying hippocampal hyperexcitability in the pathogenesis of AD is still unclear, it has been proposed to involve a deregulation of GABAergic interneurons [60,61,63] and an altered astrocytic support [64,65]. Remarkably, hippocampal A_2A_R can control the excitability of GABAergic neurons [67], the release of GABA [68,69] and the stability of GABAergic synapses [70]. A_2A_R are also located in astrocytes in the hippocampus (e.g., [71,72,73]), controlling GABA uptake [74], and astrocytic A_2A_R control neuronal excitability and memory performance [73,75,76]. These mechanistic links are in agreement with the observed upregulation of A_2A_R in synapses as well as in total membranes of the hippocampus during the presymptomatic phase of AD; however, future work is required to unveil the relative contribution of these different mechanism(s) operated by A_2A_R to control hippocampal hyperexcitability present during the presymptomatic phase of AD.

The pattern of alteration of hippocampal synaptic plasticity in our tentative presymptomatic Aβ-icv model of AD is also in agreement with the currently accepted evolution of hippocampal activity during the evolution of AD, starting with a hyperexcitability in the presymptomatic phases that gradually evolves into a hypofunction with the onset and aggravation of memory deficits [55,77,78]. The density of synaptic A_2A_R is increased before and remains increased after the onset on synaptic plasticity and memory deficits in this Aβ-icv model of AD. Furthermore, A_2A_R blockade reverted the deficits of synaptic plasticity in a manner similar to that previously reported in the normalization of abnormal synaptic plasticity in the hippocampus in different animal models of AD [8,22,32,33,41] and those of other brain diseases [24,26,42,44,79,80,81,82,83]. The effects of A_2A_R in the control of abnormal synaptic plasticity likely reflect the key role of A_2A_R in the control of memory dysfunction, as heralded by the previous observations that A_2A_R activation is sufficient to trigger memory dysfunction [27,28,29], whereas A_2A_R blockade dampens memory dysfunction in AD models [8,22,25,32,33,34,41], specifically in the currently used Aβ-icv model of AD [25,41,52]. The mechanisms underlying this beneficial effect of A_2A_R antagonists are probably related to the impact of A_2A_R on the release of glutamate [18,19], on AMPA [84] and NMDA receptors [20,21,22,85,86], as well as the ability of A_2A_R to control astrocytic function [71,72,73,74,75,76] and microglia [87,88,89,90], impacting neuroinflammation (e.g., [91,92,93]). Future work is needed to clarify the contribution of these different pools of A_2A_R to controlling these different processes known to impact synaptic plasticity and memory performance in order to begin tackling the mechanisms associated with the ability of A_2A_R blockade to normalize synaptic plasticity. These will probably involve the engagement of different heteromers containing A_2A_R (reviewed in [94]) and the coupling of A_2A_R to different transducing mechanisms, which may change with the evolution of brain diseases and the upregulation of A_2A_R (reviewed in [14]). Thus, understanding the spatiotemporal dynamics of A_2A_R signaling in different compartments—which often have opposite effects on synaptic and behavioral outputs (see [95,96,97,98])—will be critical to understand this apparently paradoxical ability of A_2A_R blockade to prevent aberrant hyperexcitability and normalize synaptic plasticity both when it is increased as well as when it is decreased. 

## 5. Conclusions

It is concluded that A_2A_R upregulation is an early event in the presymptomatic phase of AD that is maintained throughout the onset of memory deficits. This prompts consideration of the cortical A_2A_R upregulation as a putative novel biomarker of the risk of developing AD [46], and eventually as a general biomarker of neuropsychiatric diseases [99] in view of the association of A_2A_R overfunction with different neuropsychiatric diseases [99]. Moreover, the present observation that A_2A_R blockade prevents both the initial hyperexcitability during the presymptomatic phase of AD and the opposite decreased synaptic plasticity emerging with the onset of memory deficits confirms the prophylactic and therapeutic potential of A_2A_R antagonists and stresses the need to understand the different signaling mechanisms operated by A_2A_R in different cellular compartments. 

## Data Availability

The data that support the findings of this study are available from the corresponding author upon reasonable request.

## Abbreviations

A_2A_R: adenosine A_2A_ receptors; Aβ: amyloid β peptides; ACSF: artificial cerebrospinal fluid; AD: Alzheimer’s disease; EPSP: excitatory post-synaptic potential; icv: intracerebroventricular; LTP: long-term potentiation; SCH58261: 2-(2-furanyl)-7-(2-phenylethyl)-7H-pyrazolo [4,3-e][1,2,4]triazolo[1,5-c]pyrimidin-5-amine.
